# Supplementary material for: Developing a Natural Language Processing tool to identify perinatal self-harm in electronic healthcare records
Source: PLoS One. 2021 Aug 4;16(8):e0253809. doi: 10.1371/journal.pone.0253809 (PMC8336818; doi:10.1371/journal.pone.0253809)
Supplement: S1 Table — (DOCX) [file pone.0253809.s001.docx]

**S1 Table. Number of True Mentions of Self-Harm Per Service-User, Within the Reference Standard Dataset**

| **Number of "true" mentions** | **Number of Patients** |
| --- | --- |
| 0 | 123 |
| 1 | 6 |
| 2 | 5 |
| 3 | 4 |
| 4 | 4 |
| 5 | 2 |
| 6 | 0 |
| 7 | 1 |
| 8 | 3 |
| 9 | 1 |
| 10 | 1 |
| 18 | 1 |
| 23 | 0 |
| 34 | 1 |
