# Supplementary material for: Developing a Natural Language Processing tool to identify perinatal self-harm in electronic healthcare records
Source: PLoS One. 2021 Aug 4;16(8):e0253809. doi: 10.1371/journal.pone.0253809 (PMC8336818; doi:10.1371/journal.pone.0253809)
Supplement: S2 Table — (DOCX) [file pone.0253809.s002.docx]

**S2 Table.** **Macro-Averaged Pairwise Inter-Annotator Agreement**

|  | **Precision** | **Recall** | **F-score** | **Kappa** |
| --- | --- | --- | --- | --- |
| **Span** | 0.83 | 0.89 | 0.85 | N/A |
| **Polarity** | 0.96 | 0.96 | 0.96 | 0.92 |
| **Temporality** | 0.60 | 0.60 | 0.60 | 0.78 |
| **Status** | 0.66 | 0.66 | 0.66 | 0.88 |
