# Supplementary material for: Developing a Natural Language Processing tool to identify perinatal self-harm in electronic healthcare records
Source: PLoS One. 2021 Aug 4;16(8):e0253809. doi: 10.1371/journal.pone.0253809 (PMC8336818; doi:10.1371/journal.pone.0253809)
Supplement: S3 Table — (DOCX) [file pone.0253809.s003.docx]

**S3 Table.** **Macro-averaged mention-level performance**

|  | **Development set** | | | | **Test set** | | | |
| --- | --- | --- | --- | --- | --- | --- | --- | --- |
|  | **Precision** | **Recall** | **F-score** | **Kappa** | **Precision** | **Recall** | **F-score** | **Kappa** |
| **Span** | 0.97 | 0.85 | 0.90 | N/A | 0.94 | 0.81 | 0.87 | N/A |
| **Polarity** | 0.94 | 0.95 | 0.94 | 0.88 | 0.95 | 0.96 | 0.96 | 0.91 |
| **Temporality** | 0.79 | 0.78 | 0.79 | 0.57 | 0.81 | 0.82 | 0.81 | 0.62 |
| **Status** | 0.61 | 0.58 | 0.59 | 0.76 | 0.60 | 0.55 | 0.56 | 0.68 |
