# Supplementary material for: Developing a Natural Language Processing tool to identify perinatal self-harm in electronic healthcare records
Source: PLoS One. 2021 Aug 4;16(8):e0253809. doi: 10.1371/journal.pone.0253809 (PMC8336818; doi:10.1371/journal.pone.0253809)
Supplement: S1 File — (DOCX) [file pone.0253809.s004.docx]

**S1 File. List of Synonyms for Self-Harm**

| Self-harm  Self harm  Selfharm  Self-harmer  Self-harming  Self-harmed  Harm to self  Harm to herself  Harmed herself  Harmed themselves  DSH  D.S.H.  Parasuicide  Parasuicidality  Parasuicidal  Injured herself intentionally  Intentional injury  Intentional self-injury | Self-injurious behaviour  Self-mutilation  Automutilation  Mutilated herself  Mutilating herself  Attempted suicide  Suicide attempt (s)  Attempted to commit suicide  Tried to commit suicide  Tried to kill herself  Suicidal gesture  Suicidal behaviour  Admitted an overdose  Disclosed an overdose  Reported an overdose  Took an overdose  Overdosed  Overdosing  Tried to poison herself | Self-poisoning  Poisoned herself  Cut herself  Cut themselves  Reported cutting  Engaged in cutting  Made lacerations  Lacerated herself  Lacerations from self-harm  Lacerations from harming herself  Scratched herself  Slitting her wrists  Slit her wrists  Lacerate her  Burn herself  Burned herself  Self-immolation  Tried to hang herself | Attempted to hang herself  Attempted hanging  Attempted drowning  Tried to drown herself  Electrocuted herself  Tried to electrocute herself  Hit herself  Hitting herself  Self-hitting  Jumped off  Jumper from  Tried to jump |
| --- | --- | --- | --- |
