# Supplementary material for: Developing a Natural Language Processing tool to identify perinatal self-harm in electronic healthcare records
Source: PLoS One. 2021 Aug 4;16(8):e0253809. doi: 10.1371/journal.pone.0253809 (PMC8336818; doi:10.1371/journal.pone.0253809)
Supplement: S2 File — (DOCX) [file pone.0253809.s005.docx]

**S2 File. Annotation Guidelines**

**Span Annotation**

1. Only highlight the keyword not the surrounding text or whole sentence.
2. Where there are **two** mentions in a sentence, where it is implied the author means two different things, annotate them separately:

- history of self-harm and suicide attempts 🡪 here it is implied that the author views self-harm and suicide attempts as two different entities, so annotate both

1. The keyword is usually a **noun**, that is a synonym self-harm

- She has a history of self-harm in the…
- No risk of attempted suicide

1. Where the keyword is **not** a noun, highlight the noun/pronoun which it relates to:

- She has cut herself
- She used to hit her head against a wall
- X tried to jump out of a window

1. On occasion there will be mentions where the keyword is a noun, but that noun does not necessarily amount to a synonym for self-harm. In these cases, it is the *meaning* of the keyword that decides the span:

- A noun for what is suspected to be self-harm e.g. ‘she had scratches on her arm’ 🡪 annotate only the noun ‘scratches’

**Attribute Annotation**

**LEVEL 1 – STATUS - Available attributes: “relevant” or “non-relevant” or “uncertain”.**

The purpose of this level is to identify mentions that refer to an act of self-harm that has occurred, either in the past or currently.

**STATUS = RELEVANT**

Examples:

- ‘Has a history of self-harm --> *status/relevant*, since an act occurred in the past.
- ‘Took an overdose yesterday’ --> *status/relevant*, since an act occurred concurrently

Sometimes a keyword bridges two statements which have different meanings. In the example below, one part of the sentence is a statement of future risk (she would probably not take an overdose), which is irrelevant, and one part is a statement of historical self-harm (she has taken an overdose in the past). In these circumstances, err on the side of picking up events, so annotate in relation to the part that is most significant in terms of picking up acts, with current prioritised over historical.

Example:

- ‘she would probably not take an overdose as she has done this in the past’ 🡪 *status/relevant* *temporality/historical*

Have a low threshold for what is considered an act:

Example:

- ‘she had cuts…these appear to be self-inflicted…’ 🡪 *status/relevant*

**STATUS = UNCERTAIN**

If it is definitely unclear whether an act of self-harm occurred, use ‘uncertain’.

Example:

- ‘She had a bruise on her arm’ i.e. it is unclear whether this was a self-inflicted injury or not 🡪 *status/unclear*

**STATUS = NON-RELEVANT**

If an act of self-harm did not occur, annotate as ‘non-relevant’. This attribute includes: suicidal ideation; risk assessment statements of future self-harm risk; command hallucinations.

Examples:

- ‘denied self-harming’ 🡪 by definition if you are saying something hasn’t happened, it is not an act 🡪 *status/non-relevant*
- ‘heard voices telling her to harm herself’ 🡪 hallucination not an act 🡪 *status/non-relevant*
- If third-party self-harm is documented, one could argue they technically fulfil all the criteria of relevant/historical/positive, but these mentions are clearly not relevant to the study. These should be coded as *status/non-relevant*.

**LEVEL 2 – TEMPORALITY – Available attributes: “current” or “historical” or “uncertain”**

**TEMPORALITY = HISTORICAL**

Mentions of "no history of/has a history of" should be coded as historical.

Examples:

- ‘took an overdose in 1996’ 🡪 *status/relevant temporality/historical*
- ‘has a history of taking overdoses in the past’ 🡪 *status/relevant temporality/historical*
- ‘no history of’ 🡪 although it's a current assessment of the fact this person has not self-harmed, it references to looking into the past and the acts (had they happened) would have happened in the past 🡪 *status/non-relevant temporality/historical*

**TEMPORALITY = CURRENT**

Events which occur within one month of the document will be coded as current.

Example:

- ‘she self-harmed two weeks ago’ 🡪 *status/relevant temporality/current*

Mentions involving ongoing chronicity should be coded as current, as they are still ongoing so imply recency

Example:

- ‘chronic thoughts of self-harm - she usually manages them by...’ 🡪 *status/non-relevant temporality/current*

When there is a statement of *future* risk or something pertaining to future, code as current

Example:

- ‘the risk of future self-harm is high’ 🡪 *status/non-relevant temporality/current*

**TEMPORALITY = UNCERTAIN**

Events where it is unclear whether the event happened in the recent past (i.e. an event 3 months ago would still have been within the perinatal period for a women who is nine months pregnant) should be annotated as ‘current’ but the coder should make a comment within e-Host to flag it.

**LEVEL 3 – POLARITY – Available attributes: “positive” or “negative”**

The purpose of this level is to identify negations.

The default will be set at positive.

**POLARITY = NEGATIVE**

Examples:

- ‘Denied self-harm’ 🡪 *status/non-relevant temporality/current polarity/negative*
- ‘No history of self-harm’ 🡪 *status/non-relevant temporality/historical polarity/negative*

Occasionally negations will be indicated by symbols. The meaning of what is documented should be annotated.

Example:

- ‘Suicide attempts: X’ 🡪 *status/non-relevant temporality/current polarity/negative*

Sometimes there will be a linguistic negation but the purpose of the negation is to convey that an act did take place. In this case, annotate according to the meaning, not just the fact a negation is present:

Example:

- ‘she has not previously attempted suicide since 2006’ 🡪 *status/relevant temporality/historical polarity/positive*

============================================================

**Worked Examples**

- Another person's opinion/view of the self-harm: “Her mother thought the overdose was..” 🡪 *status/relevant temporality/current polarity/positive*
- Statement of future risk: “it is likely she may self-harm in future” 🡪 *status/non-relevant temporality/current polarity/positive*
- Where there is a typo e.g. "suicidal attempts" - highlight the whole phrase i.e. the meaning of what the writer was trying to say (“suicide attempts”)
